# Evaluation of the Association between the Risk of Central Retinal Artery Occlusion and the Concentration of Environmental Air Pollutants

**DOI:** 10.3390/jcm8020206

**Published:** 2019-02-07

**Authors:** Andrzej E. Grzybowski, Małgorzata K. Mimier

**Affiliations:** 1 Department of Ophthalmology, University of Warmia and Mazury, Al. Warszawska 30, 10-082 Olsztyn, Poland; ae.grzybowski@gmail.com; 2 Institute for Research in Ophthalmology, Poznan, Gorczyczewskiego 2/3, 61-553 Poznan, Poland; 3 Department and Clinic of Ophthalmology, Wroclaw Medical University, Ul. Borowska 213, 50-556 Wroclaw, Poland

**Keywords:** central retinal artery occlusion, air pollution, pollution emission

## Abstract

The purpose of the retrospective, population-based study was to assess the relationship between the risk of central retinal artery occlusion (CRAO) and the level of air pollutants. This study identified 2.272 cases of newly diagnosed CRAO registered in the Polish National Health Service database. The study authors gathered hourly ambient concentrations of particulate matter—PM 2.5, PM 10, benzene, carbon monoxide, nitrogen dioxide, ozone, and sulfur dioxide from pollution monitoring stations. Data on average daily temperature and atmospheric pressure were also obtained. In the statistical analyses, single- and multi-factor Poisson negative binomial regression models were carried out, controlling also for ambient temperature and atmospheric pressure with seasonality set at a level of 4. This study has shown a positive association between CRAO onset and short-term, daily changes in PM 10, NO_2_, SO_2_, O_3_, and CO concentrations, as well as with air temperature, in the days preceding the diagnosis.

## 1. Introduction

Central retinal artery occlusion (CRAO) is the blockage of the central retinal artery caused by thrombotic or embolic material. The embolic occlusion can be caused by cholesterol, calcium, and platelet-fibrin deposits. The formation of the thrombus is associated with atherosclerosis, inflammation, hypercoagulability, collagen vascular disease, atrial fibrillation, and oral contraceptives [1,2].

The risk factors of CRAO are cardiovascular and cerebrovascular diseases, such as systemic hypertension, hyperlipidemia, carotid artery disease, coronary artery disease, diabetes mellitus, as well as obesity and nicotinism. There is a slight male preponderance for the occurrence of CRAO compared to females. Patients diagnosed with CRAO have a life expectancy of 5.5 years compared to 15.4 years for age-matched non-CRAO patients, which tends to reflect the fact that CRAO is a multifactorial disease that has common risk factors with other cerebrovascular entities [2]. It has been suggested that short-term exposure to air pollutants and changes in meteorological parameters are associated with cardioembolic strokes [3,4]. Recent discoveries also imply a positive impact of air pollution on the occurrence of CRAO [5].

Retinal hypoperfusion can lead to ischemia of the retina and optic nerve head, causing sudden, irreversible vision loss. Because of its vision-threatening character, early diagnosis and management are crucial to protecting the retina from cell damage and blindness.

New data from World Health Organization (WHO) shows that 9 out of 10 people breathe air containing high levels of pollutants. WHO estimates that around 7 million people die every year from exposure to fine particles in polluted air that penetrate deep into the lungs and cardiovascular system, causing strokes, heart disease, lung cancer, chronic obstructive pulmonary diseases and respiratory infections, including pneumonia and more. More than 90% of air pollution-related deaths occur in low- and middle-income countries, mainly in Asia and Africa, followed by low- and middle-income countries of the Eastern Mediterranean region, Europe and the Americas. Due to the extremally bad air quality in Poland, there is a need, particularly there, for an evaluation of the association between the risk of CRAO and the concentration of environmental air pollutants. Throughout the globe, as well as in Poland, air pollution is a significant problem and an increasing number of scientific reports indicates that it is also a major health affecting issue. The progressive implementation of the improvement of air quality worldwide makes the topic interesting in order to increase the mobilization of government and non-governmental organizations.

## 2. Materials and Methods

In this retrospective study, 2.272 cases of newly diagnosed central retinal artery occlusion registered in the Polish National Health Service (NHS) database from 1 January 2014 to 30 December 2016 were identified. The National Health Service is a state organizational unit, which oversees the financing of the health system in Poland.

Our study is a retrospective, epidemiological study, in which sources of data are publicly available and patients data was collected in such a manner that subjects cannot be identified. Because of the aforementioned reasons, in accordance with Polish regulations, IRB (in Poland—Bioethics Committee) approval was not required for this study.

The study authors included all registered cases, in the given time interval, diagnosed with a central retinal artery occlusion in Poland. The data received from the NHS contained the exact date of diagnosis of CRAO and was divided into voivodships as to where these cases occurred. No cases of reported central retinal artery occlusion from 1 January 2014 to 30 December 2016 in Poland have been excluded from the study.

CRAO was defined in accordance with the International Statistical Classification of Diseases and Related Health Problems ICD-10 with diagnostic code H34.1. In the study, the date of the patient’s registration to the health care unit, with a CRAO diagnosis, was considered the first day of the disease due to its sudden nature. The study did not collect data about the age, sex, race, or comorbidities of the patient.

The study authors gathered hourly ambient concentrations of particulate matter (PM)—PM 2.5, PM 10, benzene (C_6_H_6_), carbon monoxide (CO), nitrogen dioxide (NO_2_), ozone (O_3_), and sulfur dioxide (SO_2_) from pollution monitoring stations. The data on average daily temperature and atmospheric pressure were also obtained. The data collected from the multiple monitoring stations in the region were averaged to give a daily concentration of each air impurity in a region.

The measurements were performed via 264 monitoring stations in 16 polish voivodships. In the Lower Silesian, data from 27 measurement stations were collected, in Kuyavian-Pomeranian from 22 stations, in Lublin from 12, in Lodz from 27, in Lubusz from 7, in Lesser Poland from 22, in Masovian from 23, in Opole from 8, in Podlaskie from 6, in Subcarpathian from 12, in Pomeranian from 27, in Holy Cross from 10, in Silesian from 28, in Warmian-Masurian from 8, in Greater Poland from 16, and in West Pomeranian from 9 stations. All air quality control stations are supported by the Inspectorate of Environmental Protection, established to monitor compliance with environmental regulations, research, and to assess the state of the environment. The Inspectorate includes the Chief Environmental Inspectorate and 16 Regional Environmental Inspectorates. The Inspectorate of Environmental Protection examines the PM 10 and PM 2.5 content in the air using two complementary methods: the gravimetric (reference) method, which is recognized and used in the world as the most precise measurement method and an automatic method that has proven equivalence to the reference method.

The average daily temperature and atmospheric air pressure were collected by The Institute of Meteorology and Water Management. Measurements were performed daily. In the Lower Silesian voivodship, data from 5 meteorological stations were collected, in Kuyavian-Pomeranian voivodship from 1, in Lublin from 3, in Lodz from 3, in Lubusz from 3, in Lesser Poland from 5, in Masovian from 5, in Opole voivodship from 1, in Podlaskie voivodship from 2, in the Subcarpathian voivodship from 3, in the Pomeranian voivodship from 6, in the Holy Cross voivodship from 2, in Silesian from 4, in Warmian-Masurian voivodship from 4, in Greater Poland from 5, and in the West Pomeranian from 5 meteorological stations. Overall, atmospheric pressure and air temperature were measured daily from 56 meteorological stations. The results of the measurements were averaged for individual provinces.

The study calculated the average air pollution with individual particles on the 6th, 4th, 2nd, and 1st day before the registration of the disease (H34.1), on the day of the registration of the disease, and during the week preceding the registration of the disease.

### Statistical Analysis

For the statistical analyses, single- and multi-factor Poisson negative binomial regression models, controlling also for ambient temperature and atmospheric pressure, were carried out. The seasonality a priori was set at a level of 4 due to immanent characteristics of the natural environment in Poland’s latitude, i.e., with a temperate transitional climate and four calendar seasons.

Marginal means of the investigated variables, gauged at 0, −1, −2, −4, and −6 days before the potential onset of CRAO, were presented along with their standard errors. In addition, taking into consideration the marginal means for a week preceding the onset of CRAO, moving averages were computed for each investigated environmental condition.

A level of *p* < 0.05 was considered statistically significant. The magnitude of an effect (effect size) was expressed as an IRR (“incidence rate ratio”). For all statistical procedures, Stata^®^/Special Edition, release 14.2 (StataCorp LP, College Station, TX, USA) was employed. Multiple correlation coefficients were obtained through structural equation model estimation. The Bentler-Raykov squared multiple correlation coefficients were provided. All the investigated pollutants were correlated with one another with different strengths. Largest multiple correlation coefficients were observed for the concentration of PM 2.5 (mc2 = 0.95) (*p* < 0.001), PM 10 (mc2 = 0.97) (*p* < 0.001), and carbon oxide (mc2 = 0.75) (*p* < 0.001). The lowest multiple correlation coefficients were obtained for the concentration of ozone (mc2 = 0.23) (*p* < 0.001), and benzene (mc2 = 0.32) (*p* < 0.001).

## 3. Results

A total of 2762 cases of CRAO were analyzed, of which 887 occurred in 2014, 985 in 2015, and 867 in 2016. The number of hospitalizations due to the onset of CRAO by voivodship were not statistically significant (*p* = 0.898).

During the study period, daily average concentrations of benzene (C_6_H_6_) (*p* = 0.881), carbon monoxide (CO) (*p* = 0.830), nitrogen dioxide (NO_2_) (*p* = 0.701), ozone (O_3_) (*p* = 0.592), sulphur dioxide (SO_2_) (*p* = 0.263), fine particulate matter (PM 2.5) (*p* = 0.831), and particulate matter (PM 10) (*p* = 0.756) in ambient air did not differ significantly by voivodship. Therefore, further analyses were made from an all-Poland perspective, without division into voivodships.

In a single-factor regression analysis of investigated air pollutants and atmospheric conditions measured on the day of CRAO occurrence, statistical significance was reached for CO (IRR 1.29; CI 1.08–1.54; *p* = 0.006), NO_2_ (IRR 1.02; CI 1.02–1.03; *p* < 0.001) and PM 10 (IRR 1.01; CI 1.01–1.01; *p* = 0.018).

The multi-factor regression analysis showed different results—statistical significance was reached for air temperature (IRR 0.71; CI 0.55–0.92 *p* < 0.010), NO_2_ (IRR 1.05; CI 1.03–1.05; *p* < 0.001), SO_2_ (IRR 0.98; CI 0.97–0.99; *p* = 0.040), and PM 2.5 (IRR 0.98; CI 0.97–0.99; *p* = 0.001).

Due to immanent characteristics of the natural environment in Poland’s latitude, which is a temperate transitional climate with four calendar seasons, a regression analysis that included the effect of seasonality was performed. The results of these findings are shown in Table 1 for the single-factor regression analysis and in Table 2 for the multi-factor regression analysis.

The level of the remaining analyzed factors (PM 2.5, benzene, CO, and atmospheric pressure) on the 6th, 4th, 2nd, 1st, cumulative 7-day period prior to the onset of CRAO, and on the day of CRAO occurrence did not present any statistically significant relationships when using a regression analysis with inclusion of seasonality, with the manifestation of CRAO.

## 4. Discussion

This retrospective, nationwide study, based on the NHS data combined with air pollution data, revealed that short-term exposure to gaseous air pollutants might be positively associated with CRAO onset. We have investigated that daily changes in ambient air pollution were associated with an increased risk of CRAO. Considering other possible causes of CRAO (atherosclerotic or embolic origin, blockages composed of cholesterol, calcium or fibrocystic material or predisposing diseases, such as hypertension, valvular disease, atherosclerosis, diabetes, atrial fibrillation, coagulopathy, endocarditis, hypercoagulability, giant cell arteritis, polyarteritis nodosa, use of oral contraceptives) demonstrating the relationship between air pollutions and the CRAO sheds a light on the problem that needs to be further investigated.

The study showed a relationship between air quality and CRAO onset in the week preceding the incident, but also as our conflicting results revealed, an exact relationship between pollutants and their peak-to-CRAO onset requires more data.

In recent years, the impact of air pollution on general health has been extensively studied, focusing on more systemic diseases such as stroke and cardiovascular diseases. However, only one study investigated the impact of air pollution on the central retinal artery occlusion. The results of our study and Cheng et al. were consistent regarding exposure to SO_2_ and NO_2_. Cheng et al. investigated the risk of CRAO caused by changes in the ambient air pollution [5]. In his study, 96 newly diagnosed CRAO onset patients that were randomly selected from the Taiwan National Health Insurance Research Database were examined. Among the data about the patients’ comorbidities, the study authors gathered data regarding the same ambient parameters as the authors of this study—concentrations of PM 2.5, PM 10, NO_2_, SO_2_, and O_3_. In our study, using a multi-factor regression analysis, controlling for ambient temperature and atmospheric pressure, the authors determined the statistically significant relationship between the occurrence of CRAO and the increase in SO_2_ concentration on the day of CRAO diagnosis and the increase in NO_2_ concentration on the 6th and 4th day prior to the onset of CRAO. Cheng et al. revealed, after multi-pollutant adjustment, the increase in CRAO risk after 4 days of elevated NO_2_ levels in diabetic patients and that the risk of CRAO onset had also significantly increased in patients with hypertension and in patients 65 years old, after 1 day of elevated SO_2_ levels. The transient concentration of the other air pollutants, including PM 2.5, PM 10, and O_3_, did not significantly affect the occurrence of CRAO in the study by Cheng et al. The authors concluded that short-term exposure to aforementioned air pollutants might have a positive impact on CRAO onset. The relationship was stronger in elder patients with cardiovascular risk factors, including DM and HTN.

In a paper by Cohen et al., the study team analyzed data from the Global Burden of Diseases Study 2015 and estimated the long-time impact of the ambient air pollutant on the morbidity and mortality from ischemic heart disease, cerebrovascular disease, chronic obstructive pulmonary disease, lung cancer, and lower respiratory infections globally [6]. The study authors gathered concentrations of PM 2.5 and ozone. The results showed that exposure to ambient PM 2.5 caused 4.2 million deaths and 103.1 million lost years of healthy life in 2015, and exposure to ozone caused an additional 254,000 deaths. In the past 25 years, the global burden of disease has increased because of ambient air pollution, especially in developing countries.

Due to the common risk factors and similar pathogenesis, the increased risk of stroke in CRAO patients has been repeatedly suggested, describing CRAO as a retinal analog of a stroke [7,8]. The incidence of CRAO is approximately 1 per 100,000 people. The mean age at presentation is the early 60s. Risk factors are similar to other thromboembolic diseases and include hypertension, smoking, hyperlipidemia, diabetes, hypercoagulable states, and male gender. Approximately one-third of patients with CRAO have clinically significant ipsilateral carotid artery stenosis. A survey conducted by Park et al. analyzed 1655 cases of patients diagnosed with CRAO, investigating the risk of stroke and myocardial infarction before and after the incident [9]. The results showed that the risk is particularly increased in the first week after diagnosis of CRAO. The study authors warn and emphasize that patients with incident CRAO are at high risk of stroke development and require immediate neurological examination and risk factor screening in order to reduce mortality [10].

The exact pathogenesis of increased incidence of cardiovascular diseases and CRAO induced by heightened concentrations of air pollutants still remains uncertain. Rückerl et al. examined three subpopulations of patients to investigate the association between the inflammatory markers in blood, oxidative stress, and coagulation/fibrinolysis and air pollutions levels [11]. The study revealed elevated C-reactive protein and myeloperoxidase levels in individuals with a genetic predisposition on the detoxifying pathway. It was suggested that systemic inflammation may explain one of the possible mechanisms of CRAO onset in individuals exposed to high levels of air pollutants.

The studies conducted in China also suggest the impact of short-term exposure to PM 2.5 on the expression of cytokines involved in inflammation, coagulation, and endothelial dysfunction [12,13]. Chen et al. suggested that effects of PM 2.5 on cardiovascular diseases may be related to acute effects on cytokine expression, which may be partly mediated through effects of PM 2.5 on miRNAs that regulate cytokine expression [13]. Other mechanisms regarding vasoconstriction and hypercoagulability have also been suggested in the literature [5,14,15].

In the case-crossover study conducted by Di et al. the entire Medicare population from 1 January 2000 to 31 December 2012 was examined to estimate the association between short-term exposures to ambient fine particulate matter (PM 2.5) and ozone, and mortality in the continental United States [16]. According to the results, a 10-μg/m^3^ daily increase in PM 2.5 and a 10-ppb daily increase in warm-season ozone exposures were associated with a statistically significant increase of 1.42 and 0.66 deaths per 1 M per day, respectively. As with this study, the “case day” was defined as the date of death (in our research date of the CRAO occurring). Authors have compared daily air pollution exposure on the case day vs daily air pollution exposure on “control days”.

A multi-factor analysis showed a statistically significant negative interaction between the occurrence of CRAO and air temperature on the 6th, 4th, 1st, and cumulative 7-day period prior to the onset of CRAO. The decline in the air temperature in the entire 7-day period preceding the occurrence of CRAO has been positively associated with the diagnosis, which can be related to elevated stoking, coal burning, and use of illicit materials, thereby contributing to the increase of air pollutions and tainting of the environment. In recent years, the level of air pollution in Poland has increased significantly, especially in the winter months, which constitute a huge challenge for environmental protection institutions. In all of the European Union, 80 percent of private homes using coal are in Poland. The report, Air Quality in Europe 2018, published on 29 October, relies on data collected up to 2016 from around 2500 cities in 41 countries. The authors examined the levels of several pollutants which are dangerous for humans, including PM 10 and PM 2.5. Poland remains one of the most toxic places on the continent—especially southern Poland. According to the Chief Inspectorate from Environmental Protection, the high concentration of particulate matter in the air is a result of increased emissions from municipal sources, such as individual boilers and fireplaces (due to the temperature decrease), especially in terms of light wind and lack of precipitation.

In the opinion of the authors, analysis of air pollutants, atmospheric pressure, and air temperature in terms of CRAO occurrence, based on results obtained on the day of CRAO diagnosis, is of lower significance than the rest of the results because of unknown measurement reading times, as well as the time of patients’ admission to the hospital. 

The sample size of this study (2762 cases) is relatively large, nonetheless, the study was burdened with a number of limitations. Data regarding patients’ age, race, sex, comorbidities, lifestyle, cigarette smoking, and family history, which may significantly affect the results, as shown in the study performed by Cheng et al., were not collected. This is undoubtedly the biggest limitation of the study. Another limitation of the study was the lack of information on the exact time of CRAO occurrence. In the analysis of air pollution, the entire day of the event was taken into account. The date of the patients’ registration in the health care unit with the CRAO diagnosis was considered the first day of the disease due to its sudden nature and severe eye symptoms. However, this assumption may be misleading and could impact the results, as the affected patients might not have sought medical attention immediately after the onset of CRAO. Authors included all CRAO cases from Polish NHS. The diagnosis of CRAO can be repeatedly put into the health system. There might be a chance of follow-up patients in the study subjects. Moreover, the study authors did not compare the obtained data with a control group and control period of time to ensure the validity of the results. The data on air pollutants, air pressure, temperature, and the number of patients with CRAO diagnosis were averaged by voivodship, which is a relatively large area (from 35,558.47 km^2^ (Masovian) to 9411.87 km^2^ (Opole voivodship)). As statistical analysis has shown, the daily average concentrations of air pollutants did not differ significantly by voivodship, which means the results obtained were applicable to the entire country.

## 5. Conclusions

It should be emphasized that the paper does not sign a causal link between the risk of CRAO and the concentration of environmental air pollutants. These results only demonstrated a positive association between daily changes in PM 10, NO_2_, SO_2_, O_3_, and CO concentrations, as well as with air temperature in the days preceding the diagnosis and CRAO onset. Among the other known causes, air pollution can have an impact on the pathogenesis of CRAO. Knowing the aforementioned risk factors, we should strive to reduce them in order to minimize the risk of vision-threatening diseases, such as CRAO. This study points out that air pollution may cause blindness, therefore, the fight against pollution should be extremely important, especially for developing countries struggling with high levels of air pollutants. Changes in the policy of air quality management, focused especially on the major sources of air pollution, including coal burning, excessive road transport, and vehicular emissions, may prevent the development of CRAO. A large, randomized study that is able to minimize the limitations of this study is crucial to confirm the positive association between air pollution and CRAO onset and to create management guidelines, which nowadays are necessary given the increase in the concentration of pollutants.

## Figures and Tables

**Table 1:** The statistically significant relationships between the concentration of the air pollutants and the onset of central retinal artery occlusion using single factor regression analysis, controlling for ambient temperature and atmospheric pressure, with seasonality set at a level of 4.

Lag	Factor	IRR *	*p*-Value	95% CI
−6 days	NO_2_ (μg/m^3^)	1.02	<0.001	1.01–1.02
	PM 10 (μg/m^3^)	1.01	=0.016	1.01–1.01
	Air temperature	0.99	=0.029	0.99–0.99
−4 days	NO_2_ (μg/m^3^)	1.01	=0.001	1.01–1.02
−2 days	O_3_ (μg/m^3^)	1.01	=0.005	1.01–1.01
−1 day	NO_2_ (μg/m^3^)	0.99	=0.001	0.98–0.99
	O_3_ (μg/m^3^)	1.01	=0.028	1.01–1.01
−1 week (average)	NO_2_ (μg/m^3^)	1.01	=0.028	1.01–1.02
The day of CRAO onset	CO (mg/m3)	1.44	<0.001	1.20–1.73
	NO_2_ (μg/m^3^)	1.02	<0.001	1.01–1.02
	O_3_ (μg/m^3^)	0.99	<0.001	0.99–0.99
	SO_2_ (μg/m^3^)	1.01	=0.028	1.01–1.02
	PM 10 (μg/m^3^)	1.01	=0.008	1.01–1.01

* IRR stands for incidence rate ratio (a single-factor mixed-effects Poisson negative binomial regression was performed for each separate factor with voivodship as a random intercept. Seasonality was set at a level of 4, as stated above).

**Table 2:** The statistically significant relationship between the concentration of the air pollutants and the onset of CRAO using multifactor regression analysis, controlling for ambient temperature and atmospheric pressure, with seasonality set at a level of 4.

Lag	Factor	IRR *	*p*-Value	95% CI
−6 days	Air temperature	0.76	=0.025	0.60–0.97
	NO_2_ (μg/m^3^)	1.02	<0.001	1.01–1.02
−4 days	Air temperature	0.78	=0.042	0.61–0.99
	NO_2_ (μg/m^3^)	1.01	=0.003	1.01–1.02
	O_3_ (μg/m^3^)	1.01	<0.001	1.01–1.01
−2 days	O_3_ (μg/m^3^)	1.01	<0.001	1.01–1.01
−1 day	Air temperature	0.77	=0.031	0.61–0.98
	NO_2_ (μg/m^3^)	0.99	<0.001	0.98–0.99
	O_3_ (μg/m^3^)	1.01	<0.001	1.01–1.01
−1 week (average)	Air temperature	0.74	=0.026	0.57–0.96
	O_3_ (μg/m^3^)	1.01	<0.001	1.01–1.01
The day of CRAO onset	CO (mg/m^3^)	1.46	<0.001	1.21–1.75
	NO_2_ (μg/m^3^)	1.02	<0.001	1.02–1.03
	O_3_ (μg/m^3^)	0.99	<0.001	0.99–0.99
	SO_2_ (μg/m^3^)	1.01	=0.031	1.01–1.02
	PM 10 (μg/m^3^)	1.01	=0.007	1.01–1.01

The regression equations were standard controlled for air temperature, atmospheric pressure, variability over time and voivodships—taking into account the mutual correlation between the above-mentioned independent variables. The temperature itself was not controlled for itself. * IRR stands for incidence rate ratio (* a multifactor mixed-effects Poisson negative binomial regression was performed, controlling for ambient temperature and atmospheric pressure with voivodship as a random intercept. Seasonality was set at a level of 4, as stated above).

## References

[1] Varma D.D., Cugati S., Lee A.W., Chen C.S. (2013). A review of central retinal artery occlusion: Clinical presentation and management. Eye.

[2] Agarwal N., Gala N.B., Karimi R.J., Turbin R.E., Gandhi C.D., Prestigiacomo C.J. (2014). Current endovascular treatment options for central retinal arterial occlusion: A review. Neurosurg. Focus..

[3] Chung J.W., Bang O.Y., Ahn K., Park S.S., Park T.H., Kim J.G., Ko Y., Lee S., Lee K.B., Lee J. (2017). Air pollution is associated with ischemic stroke via cardiogenic embolism. Stroke.

[4] Magalhães R., Silva M.C., Correia M., Bailey T. (2011). Are stroke occurrence and outcome related to weather parameters? Results from a population-based study in Northern Portugal. Cerebrovasc. Dis..

[5] Cheng H.C., Pan R.H., Yeh H.J., Lai K.R., Yen M.Y., Chan C.L., Wang A.G. (2016). Ambient air pollution and the risk of central retinal artery occlusion. Ophthalmology.

[6] Cohen A.J., Brauer M., Burnett R., Anderson H.R., Frostad J., Estep K., Balakrishnan K., Brunekreef B., Dandona L., Feigin V. (2017). Estimates and 25-year trends of the global burden of disease attributable to ambient air pollution: An analysis of data from the Global Burden of Diseases Study 2015. Lancet.

[7] Hayreh S.S., Podhajsky P.A., Zimmerman M.B. (2009). Retinal artery occlusion: Associated systemic and ophthalmic abnormalities. Ophthalmology.

[8] Lee J., Kim S.W., Lee S.C., Kwon O.W., Kim Y.D., Byeon S.H. (2014). Co-occurrence of acute retinal artery occlusion and acute ischemic stroke: Diffusion-weighted magnetic resonance imaging study. Am. J. Ophthalmol..

[9] Park S.J., Choi N.K., Yang B.R., Park K.H., Lee J., Jung S.Y., Woo S.J. (2015). Risk and risk periods for stroke and acute myocardial infarction in patients with central retinal artery occlusion. Ophthalmology.

[10] Youn T.S., Lavin P., Patrylo M., Schindler J., Kirshner H., Greer D.M., Schrag M. (2018). Current treatment of central retinal artery occlusion: A national survey. J. Neurol..

[11] Rückerl R., Hampel R., Breitner S., Cyrys J., Kraus U., Carter J., Dailey L., Devlin R.B., Diaz-Sanchez D., Phipps R. (2014). Associations between ambient air pollution and blood markers of inflammation and coagulation/fibrinolysis in susceptible populations. Environ. Int..

[12] Chen R., Zhao A., Chen H., Zhao Z., Cai J., Wang C., Yang C., Li H., Xu X., Li T. (2015). Cardiopulmonary benefits of reducing indoor particles of outdoor origin: A randomized, double-blind crossover trial of air purifiers. J. Am. Coll. Cardiol..

[13] Chen R., Li H., Cai J., Wang C., Lin Z., Liu C., Niu Y., Zhao Z., Li W., Kan H. (2018). Fine particulate air pollution and the expression of microRNAs and circulating cytokines relevant to inflammation, coagulation, and vasoconstriction. Environ. Health Perspect..

[14] Christophersen D.V., Jacobsen N.R., Jensen D.M., Kermanizadeh A., Sheykhzade M., Loft S., Vogel U., Wallin H., Møller P. (2016). Inflammation and vascular effects after repeated intratracheal instillations of carbon black and lipopolysaccharide. PLoS ONE.

[15] Finch J., Conklin D.J. (2016). Air pollution-induced vascular dysfunction: Potential role of endothelin-1 (ET-1) system. Cardiovasc. Toxicol..

[16] Di Q., Dai L., Wang Y., Zanobetti A., Choirat C., Schwartz J.D., Dominici F. (2017). Association of short-term exposure to air pollution with mortality in older adults. JAMA.

